# Heavy shoulder strengthening exercise in people with hypermobility spectrum disorder (HSD) and long-lasting shoulder symptoms: a feasibility study

**DOI:** 10.1186/s40814-020-00632-y

**Published:** 2020-07-10

**Authors:** Behnam Liaghat, Søren T. Skou, Uffe Jørgensen, Jens Sondergaard, Karen Søgaard, Birgit Juul-Kristensen

**Affiliations:** 1 Research Unit for Musculoskeletal Function and Physiotherapy, Department of Sports Science and Clinical Biomechanics, University of Southern Denmark, Odense, Denmark; 2 Department of Physiotherapy and Occupational Therapy, Næstved-Slagelse-Ringsted Hospitals, Slagelse, Denmark; 3 Orthopedic Research Unit, Odense University Hospital, University of Southern Denmark, Odense, Denmark; 4 Research Unit of General Practice, Faculty of Health Science, University of Southern Denmark, Odense, Denmark; 5 Research Unit of Physical Activity and Health in Work Life, Department of Sports Science and Clinical Biomechanics, University of Southern Denmark, Odense, Denmark; 6 Department of Clinical Research, University of Southern Denmark, Odense, Denmark

**Keywords:** Hypermobility, Hypermobility spectrum disorder, Shoulder, Strength, WOSI

## Abstract

**Background:**

People with hypermobility spectrum disorder (HSD) are in great risk of experiencing shoulder symptoms, but evidence for treatment is sparse. Therefore, the objective was to evaluate the feasibility of 16-week shoulder strengthening programme for improving shoulder strength and function in people with HSD and shoulder symptoms for more than 3 months to inform a future randomised controlled trial (RCT).

**Methods:**

Twelve participants (11 females, 39.3 ± 13.9 years) with HSD and shoulder instability and/or pain for more than 3 months underwent a 16-week heavy shoulder strengthening exercise programme three times weekly using exercises targeting scapular and rotator cuff muscles. Primary outcomes were pre-defined research progression criteria including recruitment rate (acceptable, 6 participants/month), assessment duration (acceptable: < 120 min), participant retention (acceptable: > 80% complete intervention), training adherence (acceptable: > 75% adhere to > 36 training sessions) and adverse events (acceptable: minor events with no participants discontinuing the study), besides participant and physiotherapist feedback. Secondary treatment outcomes were assessed using the Western Ontario Shoulder Instability Index (WOSI, 0–2100 better to worse), self-reported pain, kinesiophobia and fatigue, isometric shoulder strength, besides clinical tests for instability, hypermobility, laxity, and proprioception.

**Results:**

Recruitment rate was 5.6/month, assessment duration (mean ± SD) 105 ± 9 min, retention 100%, adherence 83%, and four participants experienced short-lasting soreness or pain. Participant feedback was positive, and physiotherapists found the intervention relevant and applicable to the population. The WOSI total score showed an improvement by 51% (mean ± SD, points: baseline 1037 ± 215; Follow up 509 ± 365; mean change (95% CI), − 528 (− 738, − 318)), and participants reported reduced pain, kinesiophobia and fatigue. Shoulder strength measurements improved by 28–31% (mean change (95% CI), Nm/kg: scaption 0.51 (0.23, 0.78); internal rotation 1.32 (0.70, 1.95) and external rotation 0.89 (0.37, 1.40)), and clinical tests indicated decreased shoulder laxity/instability.

**Conclusions:**

The shoulder strengthening exercise programme was feasible and safe for people with HSD and long-lasting shoulder symptoms. A future RCT, with an improved recruitment strategy, will demonstrate whether the exercise programme is also effective in improving symptoms and muscle-tendon function in this population.

**Trial registration:**

ClinicalTrials.gov: NCT03547570. Registered on May 3, 2018.

## Background

Generalised joint hypermobility (GJH), characterised by the capability to move the joints beyond normal range of motion [1], has an estimated prevalence of 30% in the Danish population, but the prevalence varies between 2% and 57% in adults depending on the diagnostic criteria used and population investigated [1–3].

Approximately 80% of people with GJH experience shoulder symptoms [4–6], and most display strength deficits, altered muscle activity [4, 7], instability due to repeated episodes of joint subluxations, functional deficits, and chronic pain [8–10]. GJH with such symptoms has recently been classified as hypermobility spectrum disorder (HSD) [11]. Several studies have found that people with HSD combined with shoulder symptoms report lower health-related quality of life than healthy controls [5, 6, 12].

Evidence for clinical management of people with HSD combined with shoulder symptoms is limited [13, 14]. Current evidence, based primarily on uncontrolled studies without long-term follow up and poorly described exercise programmes, demonstrates that people with HSD and shoulder symptoms including multidirectional instability benefit significantly from exercise-based treatment with the aim of targeting active elements which are involved in shoulder stability [4, 15–18]. However, most studies fall short of recommending specific types of exercise as superior to general advice [15–17]. Current guidelines recommend stability exercises and advice about joint protection, but evidence for these recommendations is sparse and based on theoretical ideas rather than being scientifically proven [13, 14]. From a physiological perspective, mechanical loading (e.g. heavy strengthening exercise) is known to increase muscle strength and tendon stiffness [19, 20], which seems to be a relevant treatment of HSD [21, 22]. However, the feasibility, safety, and effectiveness of heavy strengthening exercises in adults with HSD and shoulder symptoms are uninvestigated.

Therefore, the objective was to evaluate the feasibility of a 16-week progressive heavy shoulder strengthening exercise programme in people with HSD and long-lasting shoulder symptoms for more than 3 months to inform a future randomised controlled trial (RCT). Primary outcomes included pre-defined research progression criteria (participant recruitment and retention, duration of the collection of outcome measures, adherence to the exercise programme, and adverse events), besides participant and physiotherapist feedback, and a range of secondary self-reported outcomes and objective measurements.

## Methods

### Study design

This feasibility study was designed to evaluate research progression criteria in preparation of a definitive parallel-group RCT. However, because most of these criteria except the recruitment rate were related to the experimental intervention alone, no comparator or randomisation was used. No blinding was applied in follow-up measurements, but the principal investigator, who was also the outcome assessor, did not have access to baseline values until after completing follow-up assessments. Reporting was conducted according to the CONSORT statement extension to randomised pilot and feasibility trials [23] (Additional file 1), with research progression criteria based on a traffic light system of green (continue without changes), amber (apply changes to improve study design) and red (no RCT unless major changes are applied) [24]. The study complied with principles of the Declaration of Helsinki, was approved by the Regional Committees on Health Research Ethics for Southern Denmark (S-20170066), and prospectively registered in clinicaltrials.gov (NCT03547570).

### Participants

Males and females aged between 18 and 65 years were included provided they had generalised HSD (G-HSD) using the Beighton score cut off ≥ 5/9 for females up to the age of 50 years and ≥ 4/9 for females above 50 years and males [25], or historical HSD (H-HSD) if the Beighton score was 1 point below the age and sex-specific cutoffs, and the 5-part questionnaire (5PQ) was positive (≥ 2/5 positive items), plus having minimum one secondary symptomatic musculoskeletal manifestation [1, 11, 25]. Musculoskeletal manifestations were defined as self-reported (i) shoulder pain for minimum 3 months, (ii) recurrent dislocations or (iii) atraumatic instability. Atraumatic instability was defined as a minimum of three atraumatic shoulder dislocations, a history of at least two atraumatic dislocations in two different joints (a minimum of one in the shoulder), or medically confirmed atraumatic instability in at least two joints (a minimum of one in the shoulder) [25].

Exclusion criteria were systemic inflammatory rheumatic diseases, connective tissue diseases (e.g. Marfans, Stickler’s or Loeys-Dietz syndromes), Ehlers-Danlos syndrome (except hypermobile type), neurological diseases, pregnancy or childbirth and/or shoulder surgery within the past year, referred pain from the cervical spine on reasonable clinical suspicion by either the general practitioner (GP) or physiotherapist, and inability to speak and/or understand Danish.

Participants were recruited from April to July 2018 in primary care by GPs and physiotherapy clinics from the Region of Southern Denmark, who received information about the project and education about how to identify eligible participants. Participants were asked to answer a pre-screening electronic questionnaire including the 5PQ [26] and questions about shoulder symptoms through the web-based Research Electronic Data Capture (REDCap) [27]. The principal investigator contacted potentially eligible participants to schedule a final screening. Participants would then receive a confirmation of their scheduled appointment and detailed project information by email. At a physical meeting, the primary investigator gave oral information and performed physical screening using the Beighton tests. An informed written consent was obtained before enrolment, and the rights of participants were protected. The exercise intervention was performed at a physiotherapy clinic close to the participant’s home.

### Intervention

**Fig. 1:**
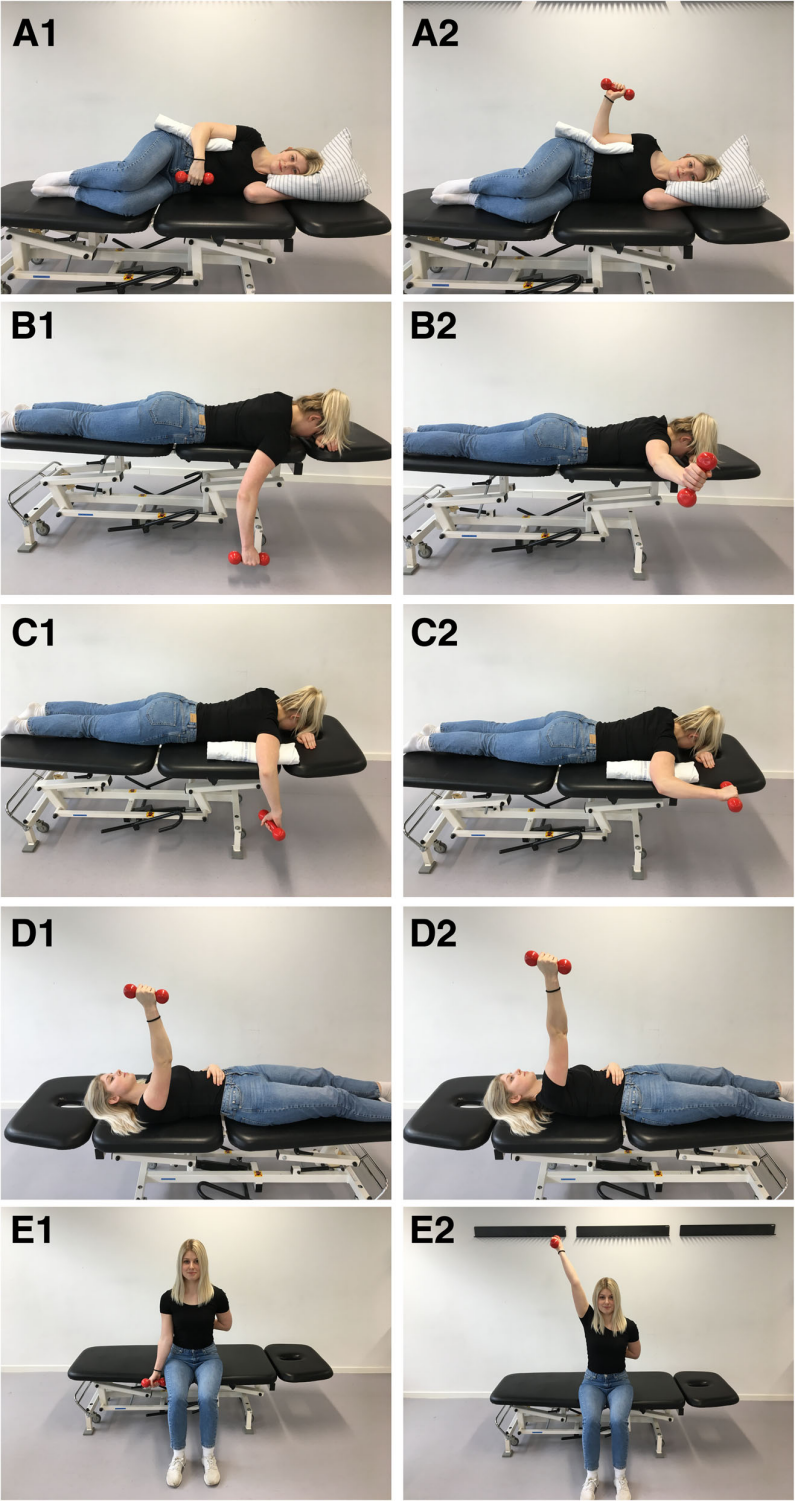
The exercise programme included five exercises targeting scapular and rotator cuff muscles. A1-A2: sidelying external rotation (ER) in neutral, B1-B2: prone horizontal abduction, C1-C2: prone ER in 90° of shoulder abduction, D1-D2: supine scapular protraction, and E1-E2: seated shoulder elevation in the scapular plane

The intervention was standardised and described according to the template for intervention description and replication (TIDieR) checklist [28] (Additional file 2), the Consensus on Exercise Reporting Template (CERT) [29] and included a thorough mechano-biological description as recommended by Toigo and Boutellier [30] (Additional file 3). Participants received 16 weeks of heavy shoulder strengthening exercise programme, supervised by a physiotherapist twice weekly and self-managed once weekly. 5-repetition maximum (RM) strength tests were carried out at first session to estimate 10 RM using Brzycki’s formula [31]. Weeks 1–3 were characterised as a familiarisation period with three sets of 50–90% of 10 RM. Weeks 4–9 included three sets of 10 RM, and from Weeks 10–15, the exercise intensity was four sets of 8 RM [22, 32]. A tapering period was applied in Week 16 to allow for anabolic response prior to follow-up assessment [32]. The load was increased whenever the participant could complete more than the pre-defined repetitions for all sets with acceptable symptoms below 5/10 on the numerical pain rating scale (NPRS) and good movement quality defined as no glenohumeral subluxation and without producing obvious scapula dyskinesis compared to unloaded movement [33]. The exercise programme included five exercises targeting scapular and rotator cuff muscles (Fig. 1) [34, 35].

Five physiotherapists (1, 3, 11, 16, and 18 years of practicing experience) had undergone a 3-h theoretical and practical education programme supported with a manual with detailed exercise instructions and the option to contact the principal investigator with questions. The same physiotherapist supervised the participant throughout the intervention period, and when necessary, another physiotherapist from the same clinic acted as stand-in. In vacation periods, participants were encouraged to self-exercise three times weekly with adjustable dumbbells provided by the project and an exercise manual written in layman’s terms.

### Outcomes

The feasibility study used a mixed outcome approach based on primary outcomes including pre-defined research progression criteria (Table 1), qualitative feedback from participants and physiotherapists, and a range of secondary self-reported outcomes and objective measurements to cover most aspects of potential benefits and identify outcomes which were responsive to the intervention. General demographic information was also obtained and included sex, age, weight, height, civil status, educational level, employment status, disease history, and physical activity level (high, moderate, or low) using the short version of International Physical Activity Questionnaire (IPAQ) [46, 47].

**Table 1:** Research progression criteria for continuing to the definitive randomised controlled trial

Outcome	Green	Amber	Red
Participant recruitment	Inclusion rate of one participant per general practitioner or physiotherapist every month (approximately *n* = 6–8/month)	(*n* < 6 after first month). If recruitment rate falls behind, screening logs and reasons for exclusion will be explored after the first month to adjust eligibility criteria.	No recruitment after 2 months
Completion of the outcome measures	Mean < 120 min to complete all objective outcome measures, and that at least 67% of participants found the duration acceptable.	Between 121 and 150 min, or only 50–66% of participants found the duration acceptable.	> 150 min or < 50% of participants found the duration acceptable
Participant retention	10 or more participants show up at 16-week follow up	Only 6–9 participants show up at 16-week follow up.	Below 6 participants show up at 16-week follow up.
Adherence to exercise intervention	Minimum 75% of participants adhering to at least 75% of exercise sessions.	Only 50–75% of participants adhering to 50–75% of exercise sessions.	< 50% of participants adhering to < 50% of exercise sessions
Adverse events	No or minor adverse events with no participants discontinuing the study	Minor or serious adverse events leading to 2 or less participants discontinuing the study	Serious adverse events leading to > 2 participants discontinuing the study

Research progression criteria were based on a traffic light system of green (go), amber (amend) and red (stop) [24]. Results of these research progression criteria were evaluated by the research group, who recommended whether to proceed with the definitive randomised controlled trial, and which amendments that needed to be made before proceeding

#### Primary outcomes

Recruitment procedures were evaluated by comparing number of participants at pre-screening with participants eligible for inclusion to identify reasons for exclusion and optimise the eligibility criteria. Recruitment rate was analysed by dividing number of included participants (*n* = 12) by the number of months it took to include them (calculated from study start until the 12th participant was recruited). To evaluate duration of baseline and follow-up assessments, completion was timed, and participants were asked if they found the duration acceptable (yes/no). Participant retention was evaluated by number of participants showing up at 16-week follow up. To evaluate exercise adherence, exercise logs were completed at each session by both the participant (at home) and the physiotherapist (when supervised) covering pain before and after exercise, load and intensity, and use of pain medication. Adherence was calculated by counting number of exercise sessions completed in the exercise log, divided by 48 planned sessions, presented in percentage.

Adverse events were registered at every exercise session and at follow up, and furthermore, participants received an electronic questionnaire by email every week with questions about adverse events, pain levels, sickness absence, use of pain medication, and additional healthcare treatment. Minor adverse events covered symptom flare-up, subluxations, and post-exercise fatigue. Serious adverse events were unexpected but covered life-threatening events, disability, or permanent damage [36]. Based on these research progression criteria, the investigators would evaluate the results and decide which amendments that needed to be made.

#### Participant and physiotherapist feedback

Participants were asked to provide feedback at 16-week follow up on a custom-made questionnaire with open questions on acceptability of assessment procedures, previous treatment experience compared with heavy shoulder strengthening exercise, and feedback about the supervised sessions and potential adverse events. Physiotherapist feedback covered whether exercises were applicable to this population and their experience with using the exercise manual, including handling the progression of exercise intensity, and exercise and load modification in case of pain flare-ups or other adverse events. Both participants and physiotherapists were asked to suggest potential improvements for the study design and procedures.

#### Secondary outcomes

##### Self-reported outcomes

The most important self-reported outcome (additional file 4) was the electronical version of Western Ontario Stability Index (WOSI), which is a valid, reliable and sensitive assessment for participants with shoulder symptoms associated with instability (0 best score to 2100 worst score) [37]. Other self-reported outcomes included pain (NPRS during past 7 days) (0 no pain, 10 extreme pain) [38]; Checklist of Individual Strength (CIS), subscale fatigue (prolonged fatigue) (8 best, 56 worst) [39]; Dartmouth Primary Care Cooperative Research Network/World Organization of National Colleges, Academies and Academic Associations of General Practitioners/Family Physicians (COOP/WONCA) (functional health status) (6 good functional status, 30 poor functional status) [40, 41]; Tampa Scale of Kinesiophobia-11 (TSK-11) (perceived fear of movement) (11 low, 44 high) [42]; Global Perceived Effectiveness (GPE) (impression of recovery) (− 5 much worse, 5 much better) [43, 44]; and European Quality of life - 5 Dimensions - Three-Level Scale (EQ-5D-3L) (health-related quality of life) (index score: < 0 to 1 (full health), with anchoring of death as 0). In addition, the EQ-5D-3L includes the European Quality of life visual analogue scale (EQ-VAS) where the patient’s own health ‘today’ is rated between 0 (worst imaginable health) and 100 (best imaginable health) [45].

##### Objective outcomes

The objective measurements (additional file 5) included seated maximum isometric voluntary contraction of shoulder scaption in 45°, internal rotation (IR), and external rotation (ER) in neutral with hand-held dynamometry (IsoForce Dynometer EVO2; Medical Device Solutions AG) [46, 48]; passive shoulder range of motion using supine ER and IR with the shoulder abducted to 90° and elbow flexed to 90°, measured with a digital inclinometer (Halo, Halo Medical Devices, Subiaco, Australia) [46, 47]; and shoulder flexion proprioception (low range 55° ± 10°, mid-range 90° ± 10°, and high range 125° ± 10°) [48]. Joint mobility and laxity parameters were assessed using load and shift, sulcus sign, Gagey, apprehension, relocation, release [49], Rotés Qúerol [50], shoulder total rotation test (positive if sum of IR and ER range of motion > 180°), and the shoulder flexion test [51].

### Sample size

No sample size calculation was performed, but 12 participants were included based on the rationale for a feasibility study, regulatory considerations and statistical considerations about a precise and representable mean and variance [52].

### Data analysis

Research progression criteria were presented with descriptive statistics. Continuous data was assessed for normality (QQ-plots and histograms) and presented as mean ± standard deviation when fulfilling assumptions for normality, or as median [interquartile range] or frequency (%). Participant and physiotherapy feedback from questionnaires were reported descriptively and organised into categories related to recruitment procedures, assessment procedures, exercise intervention, intensity progression, adverse events, and perceived treatment effect for the individual participant. Changes from baseline to follow up on secondary outcomes were assessed using paired *t* tests with significance level set to 5%. All statistical analyses were performed using Stata (StataCorp. 2019. Stata Statistical Software: Release 16. College Station, TX: StataCorp LLC).

## Results

**Fig. 2:**
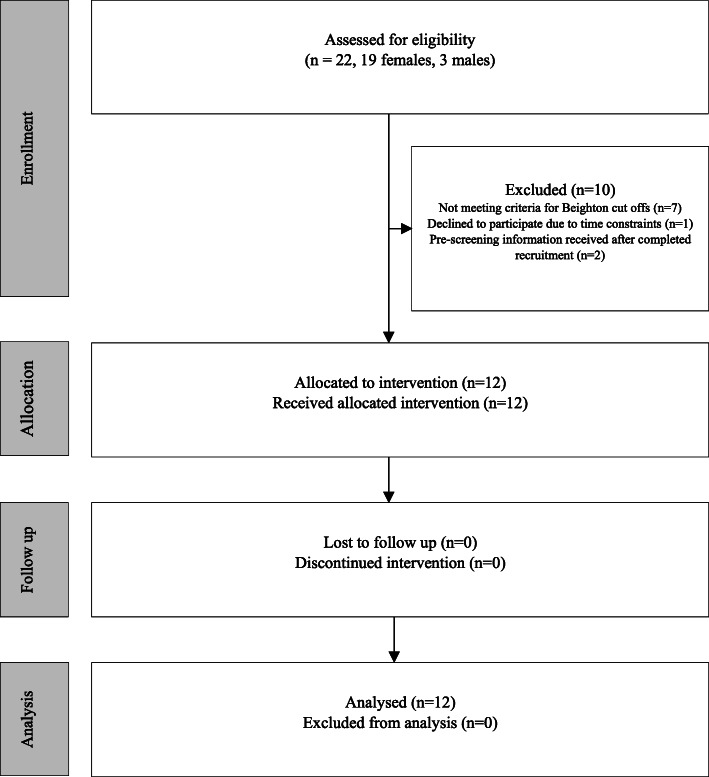
Flow diagram of participant enrolment, allocation, follow up and analysis

Twenty-two participants were assessed for eligibility from April 25 to July 11, 2018, and 12 participants (11 females) were included (Fig. 2), aged 39.3 ± 13.9 years and with median symptom duration of 36 [IQR, 15–66] months (Table 2). The main reason for exclusion was not meeting criteria for Beighton cut offs (*n* = 7). All 12 participants received the exercise intervention and were included in the analysis.

**Table 2:** Baseline demographic and clinical characteristics for participants with hypermobility spectrum disorder (HSD) and long-lasting shoulder symptoms (*n* = 12)

Characteristic	
Age, years	39.3 ± 13.9
Sex (females/males)	11/1
Height, cm	168.0 ± 7.6
Weight, kg	73.8 ± 13.5
Hypermobility spectrum disorder (HSD)	
Beighton score, 0–9	4.5 ± 1.2
5PQ, 0–5	3.4 ± 1.1
Historical HSD, *n* (%)	6 (50.0)
Generalised HSD, *n* (%)	6 (50.0)
Symptom duration, months	36 [15-66]
Shoulder pain at inclusion, *n* (%)	11 (91.7)
Previous shoulder dislocation, *n* (%)	3 (25.0)
WOSI total score, 0–2100	1037 ± 215
IPAQ short version (high, moderate, low), *n* (%)	7 (58.3), 2 (16.7), 3 (25.0)

Data are presented as mean ± standard deviation, median [interquartile range], or number (percentage)

*5PQ* 5-part questionnaire, *WOSI* Western Ontario Shoulder Instability Index, *IPAQ* The International Physical Activity Questionnaire

### Primary outcomes

Except for recruitment rate at 5.6 participants/month, level of acceptance was met for all other research progression criteria (assessment duration, participant retention, adherence, adverse events) (Table 3). During the first month, seven participants were recruited, meeting criterion for green, while recruitment rate was lower in the following months, presumably because of holidays and summer vacation.

**Table 3:** Primary outcomes in research progression criteria to inform the definitive randomised controlled trial

Research progression criteria		Evaluation
Participant recruitment rate (*n*/month)	5.6	Amber (amend)
Completion of baseline outcome measures		
Assessment duration including screening (min, mean ± SD)	105 ± 9	Green (go)
Participants answering acceptable duration (*n*, %)	12 (100.0)	Green (go)
Participant retention		
Participants who completed the follow up (*n*, %)	12 (100.0)	Green (go)
Adherence to exercise intervention		
Participants adhering to exercise programme (*n*, %)	10 (83.3)	Green (go)
Adverse events		
Minor events (*n*)	4 (33.3)	Green (go)
Serious events (*n*)	0	Green (go)
Participants discontinuing the study (*n*)	0	Green (go)

These research progression criteria were based on a traffic light system of green (go), amber (amend) and red (stop) [24]

Nine participants had a 100% completion adherence of the weekly electronic questionnaire, and the remaining three participants had completion adherence of 18.75%, 43.75% and 87.50%. Four participants reported minor adverse events: short-lasting soreness, ache, and ‘stuck’ shoulder (twice) (participant 5); headache and general soreness after exercising (participant 7); acceptable soreness (participant 10); and new pain development during handball throws (participant 12).

### Participant and physiotherapist feedback

Participants (75%) and physiotherapists (100%) were satisfied with the study design and intervention. None of the participants had been offered heavy shoulder strengthening exercise as treatment for their shoulder symptoms prior to this study, and although some participants found the heavy shoulder strengthening exercise programme demanding, they all found it relevant. Although potential challenges were holidays and sickness interfering with the 16-week long intervention, participants were able to find alternative ways to exercise during those situations. Participants reported that intensive supervision regularly was important to ensure exercises were performed correctly, and that exercise progressions were adequately applied. The physiotherapists were satisfied with the 3-h educational course about the intervention and found the exercise programme useful with satisfactory descriptions of exercise progression criteria. Four participants and all physiotherapists requested dumbbells that could be adjusted with lighter weights for precise exercise load progression.

### Secondary outcomes

#### Self-reported outcomes

**Fig. 3:**
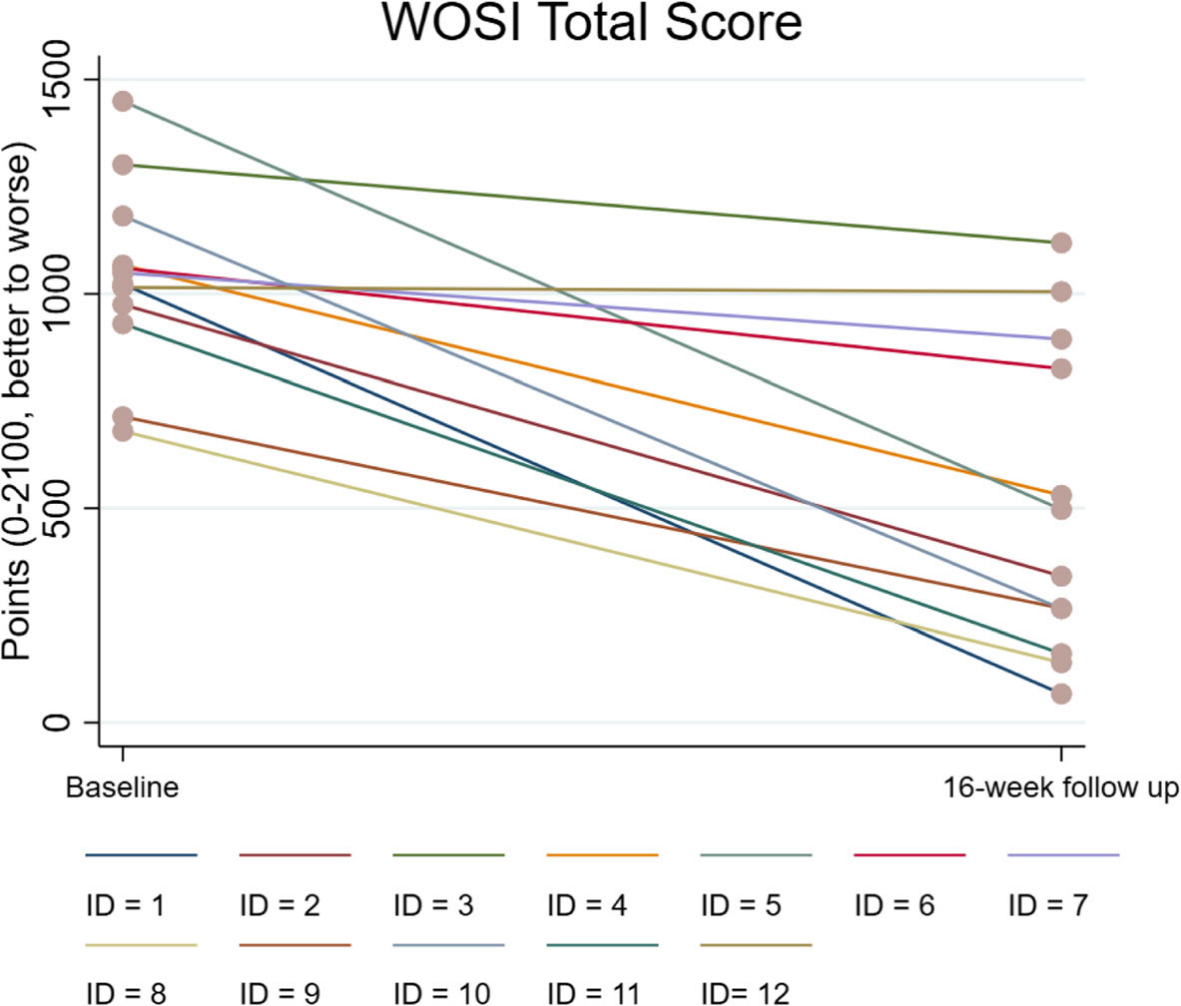
The Western Ontario Shoulder Instability Index (WOSI) total score for every participant from baseline to follow up after 16 weeks of heavy shoulder strengthening exercise programme

All participants improved in WOSI total score (on average 51%) and on all subscales (Fig. 3 and Table 4). Participants reported less pain (46–69%), decreased kinesiophobia (13%), and lower levels of fatigue (23%), and 83% had positive scores on GPE (participants 3 and 7 had 1 and 0, respectively). Overall functional status (COOP/WONCA) and general health (EQ-5D-3L) improved with 8% and 1–10%, respectively.

**Table 4:** Secondary treatment outcomes in participants with hypermobility spectrum disorder and long-lasting shoulder symptoms (*n* = 12)

	Baseline	Follow up	Within-group mean change (95% CI)	Within-group mean %—improvement
Self-reported outcome measures				
WOSI total score, 0–2100	1037 ± 215	509 ± 365	− 528 (− 738, − 318)	51
Physical symptoms, 0–1000	455 ± 104	211 ± 151	− 245 (− 337, − 152)	54
Sports/recreation/work, 0–400	228 ± 65	112 ± 97	− 117 (− 172, − 61)	51
Lifestyle, 0–400	151 ± 94	84 ± 79	− 67 (− 113, − 22)	44
Emotions, 0–300	203 ± 43	103 ± 72	− 100 (− 159, − 40)	49
Shoulder pain (NPRS) during the past 7 days				
Lowest, 0–10	1.7 ± 1.4	0.8 ± 1.1	− 0.9 (− 1.7, − 0.2)	53
Highest, 0–10	5.4 ± 2.6	2.9 ± 2.3	− 2.5 (− 3.8, − 1.2)	46
Average, 0–10	3.5 ± 1.9	1.1 ± 1.2	− 2.4 (− 3.7, − 1.2)	69
CIS, fatigue subscale, 8–56	39 ± 12	30 ± 11	− 9 (− 16, − 2)	23
COOP/WONCA, 6–30	14.3 ± 3.7	13.2 ± 3.8	− 1.2 (− 4.5, 2.1)	8
Tampa Scale of Kinesiophobia, 11–44	25.5 ± 4.4	22.3 ± 4.3	− 3.3 (− 5.7, − 0.8)	13
Global Perceived Effect, − 5 to 5	–	3.5 [3-5]	–	–
EQ-5D-3L Health status questionnaire				
EQ-VAS, 0–100	68 ± 17	75 ± 16	7 (− 7, 21)	10
Index score, < 0–1	0.80 ± 0.10	0.81 ± 0.10	0.01 (− 0.08, 0.09)	1
Objective outcome measures				
Range of motion, °				
Internal rotation passive	66.4 ± 18.8	57.5 ± 7.7	− 8.9 (− 18.8, 0.9)	13
Internal rotation active	62.3 ± 16.3	58.7 ± 8.2	− 3.5 (− 12.4, 5.3)	6
External rotation passive	100.9 ± 27.3	100.9 ± 17.0	− 0.1 (− 13.3, 13.2)	0
External rotation active	101.7 ± 22.3	103.6 ± 13.8	1.9 (− 9.3, 13.1)	− 2
Total rotation test > 180°	5 (42)	1 (8)		
Isometric shoulder strength, Nm/kg				
Scaption (*n* = 11)	1.67 ± 0.72	2.18 ± 0.76	0.51 (0.23, 0.78)	31
Internal rotation (*n* = 11)	4.57 ± 1.71	5.90 ± 1.41	1.32 (0.70, 1.95)	29
External rotation(*n* = 11)	3.17 ± 1.22	4.05 ± 1.02	0.89 (0.37, 1.40)	28
External/internal rotation ratio (*n* = 11)	0.72 ± 0.17	0.69 ± 0.10	− 0.02 (− 0.10, 0.05)	− 3
Proprioception in flexion, error (°)				
Low range, 55° ± 10°	4.9 ± 1.7	3.6 ± 1.4	− 1.2 (− 2.4, 0.0)	24
Mid-range, 90° ± 10°	4.3 ± 2.0	3.4 ± 1.5	− 0.9 (− 2.2, 0.3)	21
High range,125° ± 10° (*n* = 11)	4.3 ± 2.0	4.9 ± 3.2	0.6 (− 2.0, 3.2)	− 14
Shoulder instability and laxity tests, positive, *n* (%)				
Shoulder flexion test	1 (8.3)	2 (16.7)		
Apprehension test	6 (50.0)	4 (33.3)		
Relocation test	5 (41.7)	2 (16.7)		
Release test	4 (33.3)	2 (16.7)		
Load and shift anterior (0–3), positive 2–3	5 (41.7)	8 (66.7)		
Load and shift posterior (0–3), positive 2–3	0 (0.0)	0 (0.0)		
Sulcus sign, positive > 2 cm	0 (0.0)	0 (0.0)		
Gagey, positive > 105°	4 (33.3)	1 (8.3)		
Rotés Queról, positive > 90°	4 (33.3)	0 (0.0)		

Data are presented as mean ± SD, median [IQR], and *n* (%)

*SD* standard deviation, *IQR* interquartile range, *CI* confidence interval, *WOSI* Western Ontario Shoulder Instability Index, *NPRS* numeric pain rating scale, *CIS* Checklist Individual Strength, *COOP/WONCA* Dartmouth Primary Care Cooperative Research Network/World Organization of National Colleges, Academies and Academic Associations of General Practitioners/Family Physicians, *EQ-5D-3L* European Quality of life - 5 Dimensions – Three-Level, *VAS* visual analogue scale

#### Objective outcomes

Improvement in shoulder strength corresponded to 28–31%, and clinical tests indicated decreased shoulder laxity/instability (decreased range of motion and more participants with negative shoulder instability/laxity tests except shoulder flexion test and anterior load and shift).

## Discussion

The current study with heavy shoulder strengthening exercise in people with HSD and long-lasting shoulder symptoms more than 3 months is feasible in terms of participant retention, duration of assessments, adherence to the exercise programme, and adverse events, while recruitment rate needs to be optimised in a future RCT. Most self-reported outcomes and objective measurements showed improvements, and only few participants experienced short-lasting soreness or pain flare-ups.

The research progression criteria to inform a future RCT were based on recommendations for designing high-quality feasibility studies [53]. Although participant retention was 100%, one participant (participant 7) did not adhere to the last 4–6 weeks of exercise due to medical reasons, and another participant (participant 3) had an acute shoulder trauma unrelated to the study. This may be one reason why only these two participants did not report improvement on GPE. The high retention can be ascribed to high number of supervised sessions, where the physiotherapists motivated the participants to continue exercising, and that participants experienced the exercises as relevant and applicable for reducing their symptoms.

The duration of baseline and follow-up assessments was within the pre-defined, albeit arbitrary acceptable level of 120 min, and according to participants’ most included self-reported and objective measurements were relevant and covered important aspects of a thorough examination for their condition. To better reflect participant symptoms, which for one participant did not involve pain, NPRS could be extended to include NRS on symptoms (e.g. instability, subluxation, and laxity), and by adding the Patient Specific Functional Scale to contrast generic outcomes [54]. The shoulder flexion test (maximal passive flexion in supine) [51] and proprioception test at high range [48] were challenging to complete for participants with symptoms above 90° of shoulder flexion, and the use of these tests should be reconsidered.

Current clinical recommendations for participants with HSD advice on consulting a physiotherapist, who should prescribe exercises with low load, educate about the condition, and give advice on how to protect the joints in daily activities [13, 14, 55]. When choosing exercises for people with HSD, a general application among clinicians and researchers is to start with closed kinetic chain exercises in neutral to mid-range positions, and with no load or low loads applied to avoid excessive shear stress on the joint, before introducing more functional and full range open kinetic chain movements. Because these management considerations are contrary to the shoulder exercises offered in this study (mainly heavy load open kinetic chain movements in full range), it was imperative to register both minor adverse events and serious events in addition to exercise adherence to address potential threats to compliance. Adherence was high (83%), and based on participant feedback, maintaining supervised sessions twice a week was important for their adherence because it allowed them to be confident in performing exercises correctly and safely, besides being able to apply exercise progressions under supervised settings. Further, no serious adverse events were reported, and the reported minor adverse events (soreness and/or pain flare-up) are considered as normal short-lasting responses to heavy strengthening exercises or even to exercise in general [56, 57]. These findings support that the heavy strengthening exercise approach competes against the general understanding of how to prescribe exercise programmes for this population and could become an alternative and valuable treatment in people with HSD.

The rationale for applying heavy shoulder strengthening exercise in this population was to target the active elements that are involved in shoulder stability with the aim of impacting the cross-sectional areas of the muscles as well as the voluntary activation of the available muscle mass. Both factors would potentially in a 16-week perspective increase muscle strength and thereby the possibility to establish active support of the joint to compensate for the lack of passive joint stability in people with HSD. The observed strength improvements at 16-week follow up (without changing the ER/IR ratio) were plausibly mediated by improved neuromuscular function, primarily in the initial phase of the exercise programme, besides peripheral adaptations in the musculotendinous unit (e.g. hypertrophy and tendon stiffness [19, 20, 58]) of scapular muscles and rotator cuff muscles [30]. Muscle and tendon stiffness around the shoulder joint is difficult to examine but can be evaluated indirectly with clinical tests of shoulder laxity. Passive IR range of motion, shoulder total rotation > 180°, Gagey (inferior glenohumeral laxity), and Rotés Quérol (ER > 90°) tests indicated clinically reduced shoulder laxity. Another potential mechanism is a better ability to protect the shoulder at end range due to an improved joint awareness, which can be partly supported by the observed improvement in proprioception (sense of the relative position of one’s own parts of the body and strength of effort being employed in movement) measured at low range of shoulder flexion. Looking at the self-reported outcomes, participants improved above the minimal clinically important difference (MCID) in WOSI total score (51%; MCID 10.4–14% [59, 60]), which will be the primary outcome to evaluate the treatment effectiveness in the definitive RCT. Improvements were also observed in other relevant outcomes such as shoulder pain, kinesiophobia, and level of prolonged fatigue. Along with the questionnaires about general health status, these outcomes are important to assess when evaluating treatment effectiveness in a future RCT, and for comparison across other studies and patient populations. More in depth analyses of these secondary outcomes are outside the scope of this feasibility study, but the apparent potential benefits justify a more thorough evaluation in an RCT.

Based on the research progression criteria and findings in self-reported outcomes, objective outcome measures, and qualitative feedback, it seems feasible to proceed with an RCT if an improved recruitment strategy is applied. To ensure the inclusion of participants who are seeking professional help by their own initiative and with symptoms reaching a certain level of severity, we will involve more GPs and physiotherapy clinics in the recruitment process rather than recruiting via other channels (e.g. community advertising). Although only one out of 13 eligible participants declined to participate due to time constraints, this number is anticipated to increase in an RCT because a larger sample size is needed, why the number of people declining for various reasons will also increase, which is an incentive for improving recruitment to the absolute maximum. Another reason for declining to participate is the inherent barrier in the randomisation procedure which does not allow the participant to know their treatment in advance; however, the comparator group in the definitive RCT will receive a home-based exercise programme build on the current practice and clinical recommendations with (only) three supervised sessions, and as such this will probably not affect the recruitment rate considerably but could impair exercise adherence.

### Strengths and limitations

The strengths of the study were the standardised, transparent, and precisely described exercise programme targeting scapular and rotator cuff muscles; careful training of involved physiotherapists and supervision of participants during exercise interventions; and the pre-defined progression and evaluation criteria. Due to the inherent design of a feasibility study, methodological limitations, such as lack of a control group and inability to ensure blinding of participants and the investigator, limit conclusions that can be drawn on the effectiveness of the exercise programme. The observed improvements in secondary outcomes could be in part due to regression to the mean. Therefore, the current study findings highlight the feasibility and safety required before testing heavy shoulder strengthening exercise in an RCT.

## Conclusions

In conclusion, heavy shoulder strengthening exercise is feasible and safe for people with HSD and long-lasting shoulder symptoms for more than 3 months. A future RCT, with an improved recruitment strategy, will demonstrate whether the exercise programme is also effective in improving symptoms and decreasing shoulder laxity/instability in this population.

## Supplementary information

**Additional file 1:** CONSORT 2010 checklist of information to include when reporting a pilot or feasibility trial

**Additional file 2:** The TIDieR (Template for Intervention Description and Replication) Checklist*: Information to include when describing an intervention and the location of the information

**Additional file 3:** Exercise intervention, CERT Checklist and mechano-biological description

**Additional file 4:** Self-reported outcomes

**Additional file 5:** Objective outcomes

## Data Availability

The datasets generated and/or analysed during the current study are available from the corresponding author on reasonable request.

## Abbreviations

CIS: Checklist of Individual Strength
COOP/WONCA: Dartmouth Primary Care Cooperative Research Network/World Organization of National Colleges, Academies and Academic Associations of General Practitioners/Family Physicians
CI: Confidence interval
EQ-5D-3L: European Quality of life - 5 Dimensions - Three-Level
ER: External rotation
GJH: Generalised joint hypermobility
GPE: Global Perceived Effectiveness
HSD: Hypermobility spectrum disorder
IQR: Interquartile range
IPAQ: International Physical Activity Questionnaire
IR: Internal rotation
MCID: Minimal clinically important difference
NPRS: Numerical pain rating scale
RCT: Randomised controlled trial
RM: Repetition maximum
SD: Standard deviation
TSK-11: Tampa Scale of Kinesiophobia-11
VAS: Visual analogue scale
WOSI: Western Ontario Shoulder Instability Index
5PQ: 5-part questionnaire
