# Supplementary material for: Heavy shoulder strengthening exercise in people with hypermobility spectrum disorder (HSD) and long-lasting shoulder symptoms: a feasibility study
Source: Pilot Feasibility Stud. 2020 Jul 10;6:97. doi: 10.1186/s40814-020-00632-y (PMC7350677; doi:10.1186/s40814-020-00632-y)
Supplement: Supplementary file 3 — Additional file 3: Exercise intervention, CERT Checklist and mechano-biological description [file 40814_2020_632_MOESM3_ESM.docx]

# Additional File 3

## Exercise intervention, CERT Checklist[1]

1 Detailed description of the type of exercise equipment

The exercises were performed with adjustable dumbbells.

2 Detailed description of the qualifications, expertise and/or training

Heavy shoulder strengthening exercises provided by physiotherapist, who had approximately three hours of education about the exercise programme.

3 Describe whether exercises are performed individually or in a group

Exercises were performed individually.

4 Describe whether exercises are supervised or unsupervised; how they are delivered

Exercises were supervised twice a week and performed once a week at home (non-supervised).

5 Detailed description of how adherence to exercise is measured and reported

Participant adherence was measured with an exercise log and reported in % (100% equals full adherence corresponding to 48 exercise sessions).

6 Detailed description of motivation strategies

Participants received supervision two out of three times every week and were encouraged with an exercise manual with a detailed description of the strengthening exercise programme.

7a Detailed description of the decision rule(s) for determining exercise progression

Exercise progression was applied when the participant could perform more than the pre-defined repetitions without pain flare up or obvious signs of scapula instability.

7b Detailed description of how the exercise programme was progressed

The exercise programme was progressed by increasing load and partly by increasing volume/intensity. The load was increased whenever the participant could complete more than the pre-defined repetitions for all sets with acceptable symptoms below 5/10 on the Numerical Pain Rating Scale (NPRS) and good movement quality defined as no glenohumeral subluxation and without producing obvious scapula dyskinesis compared to unloaded movement [2].

8 Detailed description of each exercise to enable replication

The exercise programme included five exercises identified in literature to target scapular and rotator cuff muscles: a) Sidelying external rotation (ER) in neutral, b) prone horizontal abduction, c) prone ER in 90˚, d) supine scapular protraction, and e) seated shoulder elevation in the scapular plane.

9 Detailed description of any home programme component

The same programme was performed at home.

10 Describe whether there are any non-exercise components

Non-exercise components included general advice on management of load and pain.

11 Describe the type and number of adverse events that occur during exercise

Any adverse events that occurred during exercise was registered and could involve flare up in pain and episodes of subluxations or dislocations.

12 Describe the setting in which the exercises are performed

The exercises were performed in an undisturbed clinical environment and at home.

13 Detailed description of the exercise intervention

The first three weeks were a familiarisation period progressing from three sets of 50% of 10 repetition maximum (RM) in the first week to 70% in second week and 90% in the third week. The following six weeks (week 4-9) included heavy shoulder strengthening exercise with three sets of 10 RM, and from week 10-15 the exercise load was set at four sets of 8 RM. During the entire intervention period, the exercises were performed with a 3 sec concentric and a 3 sec eccentric phase without any isometric holds (Additional file 3, table 1).

14a Describe whether the exercises are generic (one size fits all) or tailored

The exercises are tailored to the individual.

14b Detailed description of how exercises are tailored to the individual

The exercises are tailored by adjusting exercise load to match the individual level of the RM.

15 Describe the decision rule for determining the starting level

The starting level was determined by determining the 1RM in each exercise at the first supervised session. Later the load was continuously adjusted to the increased capabilities of the individual participant.

16a Describe how adherence or fidelity is assessed/measured

Participant adherence is measured with an exercise log

16b Describe the extent to which the intervention was delivered as planned

The intervention was delivered as planned, with 83% of participants adhering to more than 75% of the planned exercise sessions.

| **Additional file 3, table 1**. Mechano-biological description of the exercise programme as recommended by Toigo & Boutellier [3] | | | | | | | | | | | | | |
| --- | --- | --- | --- | --- | --- | --- | --- | --- | --- | --- | --- | --- | --- |
| **Week** | **X_1_** | **X_2_** | **X_3_** | **X_4_** | **X_5_** | **X_6_** | **X_7_** | **X_8_** | **X_9_** | **X_10_** | **X_11_** | **X_12_** | **X_13_** |
| 1 | 50% 10 RM | 10 | 3 | 60 s | 3 per week | 1 week | 3 s shortening  0 s isometric  3 s lengthening | - | 60 s | No | Full ROM | 48 h | Yes |
| 2 | 70% 10 RM | 10 | 3 | 60 s | 3 per week | 1 week | 3 s shortening  0 s isometric  3 s lengthening | - | 60 s | No | Full ROM | 48 h | Yes |
| 3 | 90% 10 RM | 10 | 3 | 60 s | 3 per week | 1 week | 3 s shortening  0 s isometric  3 s lengthening | - | 60 s | No | Full ROM | 48 h | Yes |
| 4 | 10 RM | 10 | 3 | 60 s | 3 per week | 6 weeks | 3 s shortening  0 s isometric  3 s lengthening | - | 60 s | Yes | Full ROM | 48 h | Yes |
| 10 | 8 RM | 8 | 4 | 90 s | 3 per week | 6 weeks | 3 s shortening  0 s isometric  3 s lengthening | - | 48 s | Yes | Full ROM | 48 h | Yes |
| 16 | 70% 8 RM | 8 | 4 | 90 s | 3 per week | 1 week | 3 s shortening  0 s isometric  3 s lengthening | - | 48 s | No | Full ROM | 48 h | Yes |
| X_1_ load magnitude  X_2_ number of repetitions  X_3_ number of sets  X_4_ rest in-between sets  X_5_ number of sessions per week  X_6_ duration of the experimental period  X_7_ fractional and temporal distribution of the contraction modes per repetition and duration of one repetition  X_8_ Rest in between repetitions  X_9_ Time under tension (s)  X_10_ volitional muscular failure  X_11_ range of motion  X_12_ recovery time in between exercise sessions  X_13_ predefined anatomical exercise form | | | | | | | | | | | | | |

**References**

1. Slade SC, Dionne CE, Underwood M, Buchbinder R, Beck B, Bennell K, Brosseau L, Costa L, Cramp F, Cup E, Feehan L, Ferreira M, Forbes S, Glasziou P, Habets B, Harris S, Hay-Smith J, Hillier S, Hinman R, Holland A, et al. Consensus on Exercise Reporting Template (CERT): Modified Delphi Study. Phys Ther. 2016;96(10):1514-24.

2. McClure P, Tate AR, Kareha S, Irwin D, Zlupko E. A clinical method for identifying scapular dyskinesis, part 1: reliability. Journal of athletic training. 2009;44(2):160-4.

3. Toigo M, Boutellier U. New fundamental resistance exercise determinants of molecular and cellular muscle adaptations. Eur J Appl Physiol. 2006;97(6):643-63.
