# Supplementary material for: Heavy shoulder strengthening exercise in people with hypermobility spectrum disorder (HSD) and long-lasting shoulder symptoms: a feasibility study
Source: Pilot Feasibility Stud. 2020 Jul 10;6:97. doi: 10.1186/s40814-020-00632-y (PMC7350677; doi:10.1186/s40814-020-00632-y)
Supplement: Supplementary file 4 — Additional file 4: Self-reported outcomes [file 40814_2020_632_MOESM4_ESM.docx]

**Additional file 4

Self-reported outcomes**

| *WOSI* | The Western Ontario Shoulder Instability Index (WOSI) is a self-reported disease specific quality of life measurement tool for patients with shoulder instability. The WOSI consists of four subscales; (1) Physical Symptoms and pain; (2) Sport, recreation, and work function; (3) Lifestyle and social function; and (4) Emotional well-being. Each item is scored on a visual analogue scale with a line of 0-100 mm horizontally placed under each question, where the best possible score is indicated with the lowest possible score = 0 and the worst possible score is indicated with the highest possible score = 100. Twenty-one items are scored in total with a maximum of 2100 points, and a minimum of 0 points. The maximum possible score in each dimension is 1000, 400, 400, and 300 respectively. A digital Danish validated version was used (1). |
| --- | --- |
| *NPRS* | Shoulder pain was assessed by using the Numerical Pain Rating Scale (NPRS). NPRS rates pain between 0 – 10 (”no pain” to” worst imaginable pain”) (2). The pain level was measured at baseline and at 16-week follow-up for the worst, least and average pain level for the past week using an electronic questionnaire, and pain intensity before and after each training session was measured using an exercise log. |
| *CIS* | The Checklist Individual Strength (CIS) by the subscale fatigue was used to assess prolonged fatigue. The questionnaire has shown good reliability and validity and was performed at baseline and at 16-week follow-up to report the change in level of fatigue. The CIS subscale fatigue consists of eight items each scored on a 7-point Likert-scale (scores ranging from 8 to 56) with high scores indicating high levels of fatigue (3). |
| *COOP/WONCA* | To assess the change in functional health status from baseline to 16-week follow-up, the Dartmouth Primary Care Cooperative Research Network/World Organization of National Colleges, Academies and Academic Associations of General Practitioners/Family Physicians (COOP/WONCA) questionnaire was used. The questionnaire is a generic health status questionnaire for general practice patients. The questionnaire consists of six single-item measures: physical fitness, feelings (mental well-being), daily activities, social activities, besides change in health and overall health. The categories are scored from one (good functional status) to five (poor functional status) (overall score: 6 good functional status, 30 poor functional status) (4, 5). |
| *TSK-11* | The 11-item Tampa Scale of Kinesiophobia (TSK-11) was used to measure impression of change in fear of movement from baseline to 16-week follow-up. Each question is scored on a 4-point Likert-scale with 1 indicating “strongly disagree” and 4 indicating, “strongly agree”. The total score ranges from 11-44, with higher scores representing increased fear of movement (6). |
| *GPE* | To measure the patients self-rated impression of recovery at follow-up the Global Perceived Effect (GPE) scale was used. GPE measures self-rated impression of recovery since baseline assessment on a scale from -5 (much worse) to 5 (much better) (7, 8). |
| *EQ-5D-3L, including EQ-VAS* | The European Quality of life - 5 Dimensions - Three-Level (EQ-5D-3L) was used to measure change in health-related quality of life from baseline to follow-up. The generic instrument EQ-5D-3L is a classification system that comprises five dimensions (mobility, Self-care, usual activities, pain/discomfort, and anxiety/depression).  Each dimension is rated using a three-level ordinal scale as follows: 1) no problems, 2) some problems, and 3) extreme problems. This health state classification describes 243 unique health states that are often represented as vectors. From population-based valuation studies societal value sets have been derived, and when they are applied to the health status vectors, it results in a preference-based index that ranges from states worse than death (<0), to 1 (full health), anchoring dead at 0. A score of 1 indicates that the participants perceived their health at the best possible state, and a score below 0 that the participants perceived their health worse than death (9). The societal value set for Denmark was used (10).  In addition, the EQ-5D-3L includes the European Quality of life Visual Analog Scale (EQ-VAS) where own health "today" is rated on a scale from 0 (worst imaginable health) to 100 (best imaginable health). |

**References**

1. Eshoj H, Bak K, Blond L, Juul-Kristensen B. Translation, adaptation and measurement properties of an electronic version of the Danish Western Ontario Shoulder Instability Index (WOSI). BMJ Open. 2017;7(7):e014053.

2. Breivik H, Borchgrevink PC, Allen SM, Rosseland LA, Romundstad L, Hals EK, et al. Assessment of pain. Br J Anaesth. 2008;101(1):17-24.

3. Voermans NC, Knoop H. Both pain and fatigue are important possible determinants of disability in patients with the Ehlers-Danlos syndrome hypermobility type. Disabil Rehabil. 2011;33(8):706-7.

4. Bentsen BG, Natvig B, Winnem M. Questions you didn't ask? COOP/WONCA Charts in clinical work and research. World Organization of Colleges, Academies and Academic Associations of General Practitioners/Family Physicists. Fam Pract. 1999;16(2):190-5.

5. Kinnersley P, Peters T, Stott N. Measuring functional health status in primary care using the COOP-WONCA charts: acceptability, range of scores, construct validity, reliability and sensitivity to change. Br J Gen Pract. 1994;44(389):545-9.

6. Mintken PE, Cleland JA, Whitman JM, George SZ. Psychometric properties of the Fear-Avoidance Beliefs Questionnaire and Tampa Scale of Kinesiophobia in patients with shoulder pain. Arch Phys Med Rehabil. 2010;91(7):1128-36.

7. Kamper SJ, Ostelo RW, Knol DL, Maher CG, de Vet HC, Hancock MJ. Global Perceived Effect scales provided reliable assessments of health transition in people with musculoskeletal disorders, but ratings are strongly influenced by current status. J Clin Epidemiol. 2010;63(7):760-6 e1.

8. Forouzanfar T, Weber WE, Kemler M, van Kleef M. What is a meaningful pain reduction in patients with complex regional pain syndrome type 1? Clin J Pain. 2003;19(5):281-5.

9. Janssen MF, Pickard AS, Golicki D, Gudex C, Niewada M, Scalone L, et al. Measurement properties of the EQ-5D-5L compared to the EQ-5D-3L across eight patient groups: a multi-country study. Qual Life Res. 2013;22(7):1717-27.

10. Wittrup-Jensen KU, Lauridsen J, Gudex C, Pedersen KM. Generation of a Danish TTO value set for EQ-5D health states. Scand J Public Health. 2009;37(5):459-66.
