# Supplementary material for: Heavy shoulder strengthening exercise in people with hypermobility spectrum disorder (HSD) and long-lasting shoulder symptoms: a feasibility study
Source: Pilot Feasibility Stud. 2020 Jul 10;6:97. doi: 10.1186/s40814-020-00632-y (PMC7350677; doi:10.1186/s40814-020-00632-y)
Supplement: Supplementary file 5 — Additional file 5: Objective outcomes [file 40814_2020_632_MOESM5_ESM.docx]

**Additional file 5**

**Objective outcomes**

| *Range of motion (ROM), shoulder internal rotation, passive and active* | Passive and active glenohumeral internal rotation were tested in 90° degree of abduction [1, 2]. A *HALO Digital Goniometer* was placed with Velcro fittings on the dorsal, distal part of the forearm at the wrist. The participant was then instructed to lie down on an examination table in a supine position with the arm in 90° abduction in the frontal plane, 90° flexion in the elbow and the forearm in supination, so that the thumb pointed vertically. A towel was folded and laid under the upper arm, so that the elbow was at the height of the glenohumeral joint and free of the edge of the examination table. The tester used a firm pressure on the processus coracoideus to prevent the protraction of the scapula. The tester kept an eye on any compensation and/or movement in the thorax. The participant was then asked to keep the scapula retracted, so there was full contact with the base of the examination table and spina scapula.  First, three measurements of passive internal rotation were performed by the tester: with one hand over the processus coracoideus, the second hand was placed proximally on the forearm to control the movement in internal rotation. The movement was stopped at end-feel or at symptom/pain stop. The measurements were then noted by the tester.  Then, three active internal rotation were performed: the participant brought the arm in the direction of the little finger, down towards the floor as far as the participant could. The movement was stopped at end-feel or at symptom/pain stop. The measurements were then noted by the tester. |
| --- | --- |
| *Range of motion (ROM), shoulder external rotation, passive and active* | Passive and active glenohumeral external rotation were tested in 90° abduction [1, 2]. A *HALO Digital Goniometer* was placed with velcro fittings on the dorsal, distal part of the forearm at the wrist. The participant was then instructed to lie down on an examination table in a supine position, with the arm in 90° abduction in the frontal plane, 90° flexion in the elbow and the forearm in supination, so that the thumb pointed vertically. A towel was folded and laid under the upper arm, so that the elbow was at the height of the glenohumeral joint and free of the edge of the examination table. The tester used a firm pressure on the processus coracoideus to prevent extra movements. The tester kept an eye on any compensation and/or movement in the thorax. The participant was then asked to keep the scapula retracted so there’s full contact with the base of the examination table and spina scapula.   First, three passive external rotation were performed by the tester: with one hand over the processus coracoideus, the second hand had a grip on the forearm to control the movement in external rotation. The movement was stopped at end-feel or at symptom/pain stop. The measurements were then noted by the tester.   Then, three active external rotation were performed: the participant brought the arm in the direction of the thumb, down towards the floor as far as the participant could. The movement was stopped at end-feel or at symptom/pain stop. The measurements were then noted by the tester. |
| *Isometric shoulder strength, Nm/kg* | Strength measurement was performed with *Isoforcecontrol® EVO2*, which is a handheld dynamometer [2, 3]. In addition, a measuring tape, a protractor, a foam block and a stool were used. Three measurements were made in each direction of movement, and all three measurements were recorded, before calculating the average. The participant got the length of the weight lever arm measured, and this measurement was used together with the measurement to find the force development in the shoulder based on the formula:   $MVC= Power-torque=Force*torque\_arm \sim\left[ \mathrm{NM} \right]=\left[ N \right]*\left[ m \right]$  The process applied to each of the three movement directions.  ***Description of the tests*** The participant warmed up by first making ten repetitions of internal rotation, external rotation and scaption with a lightweight *Theraband* resistance band.  **Scaption:** The participant was instructed to sit on the stool with the back against the wall. The arm was kept 30 degrees from the wall in scapula's plane, measured with a goniometer. The participant was instructed to keep the arm in 45° of abduction (measured with an inclinometer). The hand was positioned with the thumb towards the ceiling. The elbow was kept completely stretched. Shoulder was kept in light retraction. Before the strap was mounted on hand, the participant was instructed (see instruction below).The dynamometer was positioned vertically under the hand and the strap was adjusted so that it was just slightly tightened, but not too tight, when the strap was positioned just proximal to the base of the thumb.  ***Measurement 1:*** *The torque-arm distance from the anterior corner of acromion to the distal edge of the strap at the base of the thumb was measured.*  *IsoForceControl® EVO2* was switched on and when ready to start, the tester pressed "Start". While calibrating the device, the participant should not put pressure on the strap. After calibration, the device beeped and it said, "*Ready to push*". Three iterations were performed in scaption, and a 60 sec break was held between each repetition. It was ensured that no compensatory movements were made during execution. If this applied, then the repetition did not count. During testing the tester cheered by saying: “*PUSH, PUSH, PUSH, PUSH”*.  **Internal rotation:** The participant was instructed to sit on the stool, positioned so that the participant sat with the side to the wall. The participant was instructed to sit with a straight back and scapula slightly retracted. The elbow was placed in the middle of the thorax with a rolled towel positioned between the upper arm and the wall. The elbow was flexed to 90° and placed about 1-2 fingers width from the thorax. The hand was positioned with the thumb towards the ceiling. Before the strap was mounted on the hand of the participant, the participant was instructed (see the instruction below). The suction cup placed on the wall so that a 90° angle was formed between the *IsoForceControl® EVO2* strap and the participant´s forearm. The strap was adjusted so that it was just slightly tightened, but not tight when the strap was positioned just proximally to the base of the thumb.  ***Measurement 2:*** *The torque-arm distance from lateral epicondyle to the distal edge of the strap at the base of the thumb was measured.*  *IsoForceControl® EVO2* was switched on and when ready to start, the tester pressed "Start". While calibrating the device, the participant should not put pressure on the strap. After calibration, the device beeped and it said, "*Ready to push*". Three iterations were performed in scaption, and a 60 sec break was held between each repetition. It was ensured that no compensatory movements were made during execution. If this applied, then the repetition did not count. During testing the tester cheered by saying: “*PUSH, PUSH, PUSH, PUSH”*.  **External rotation:** The participant was instructed to sit on the stool, positioned so that the participant sits with the opposite side to the wall. The participant was instructed to sit with a straight back and scapula slightly retracted. The elbow was placed in the middle of the thorax with a rolled towel positioned between the upper arm and the wall. The elbow was flexed to 90° and placed about 1-2 fingers width from the thorax. The hand was positioned with thumb towards the ceiling. Before the strap was mounted on the hand of the participant, the participant was instructed (see the instruction below). The suction cup was placed on the opposite wall so that a 90° angle was formed between the *IsoForceControl® EVO2* strap and the participant's forearm. The strap was adjusted so that it was just slightly tightened, but not tight when the strap was positioned just proximally to the base of the thumb.  ***Measurement 3:*** *Torque arm was used.*  *IsoForceControl® EVO2* was switched on and when ready to start, the tester pressed "Start". While calibrating the device, the participant should not put pressure on the strap. After calibration, the device beeped and it said, "*Ready to push*". Three iterations were performed in scaption, and a 60 sec break was held between each repetition. It was ensured that no compensatory movements were made during execution. If this applied, then the repetition did not count. During testing the tester cheered by saying: “*PUSH, PUSH, PUSH, PUSH”*. |
| *Proprioception in flexion* | Proprioception in flexion [4, 5] was measured with a *HALO digital goniometer*. The *HALO Digital goniometer* was positioned laterally on the overarm, just above the elbow with velcro fittings. The tester stood beside the participant and noted the angle of the shoulder after each positioning.  Shoulder flexion was divided into three levels:  Low: 55° ± 10 °  Mid: 90° ± 10 °  High: 125° ± 10°  In each test session, the participant had to reproduce 9 positions in total (3 levels and 3 test attempts of each). There was a five-second break between tests.  **Procedure:** The participant was blindfolded, standing on both legs with the arms resting along the body. The tester instructed the participant to flex the shoulder with the thumb pointing upwards/vertically. The participant was then verbally stopped when the grade number reached within the predefined shoulder angle within ± 10° (position 1). The tester then counted to three and noted the position of the arm in degrees and prompted the participant to bring the arm back to neutral and immediately thereafter recover the shoulder to its previous position (position 2).The participant was then instructed to tell the tester when he/she felt that the position had been recovered. Positions 1 and 2 were noted and the difference represented the joint position sense error-value. |
| *Shoulder flexion test* | **Explanation:** The participant was supine. The tester’s arm was placed on the lower back and the participant pressed the spine gently towards the hand and held this position.  **Placement of hands:** Tester held the arm at the wrist and elbow and lifted the arm over the head.  **Evaluation:** Could the whole humerus rest easily on the examination table? Yes or no (Nominal, dichotomous variable) [6]. |
| *Apprehension test* | **Explanation:** The participant was supine with the test-shoulder close to the edge of the examination table. The shoulder was placed in 90° abduction, and the elbow was flexed to 90°. The tester moved the shoulder into maximum external rotation.  **Placement of hands:** A hand was placed around the wrist on the participant’s hand. The other hand was positioned gently on the front of the shoulder. The elbow was supported by the tester’s thigh.  **Evaluation:** Subjective or objective presence of reluctance/resistance (apprehension) and/or pain. Scored as either positive or negative (Nominal, dichotomous variable) [7]. |
| *Relocation test* | **Explanation:** From the final position of the apprehension test, the head of the humerus was gently pushed into a posterior direction of the tester.  **Placement of hands:** The fifth finger of the tester was placed close to the lateral part of the participant’s acromion with the wrist of the tester positioned anterior on the caput humeri.  **Evaluation:** Reduction of apprehension symptoms and/or pain. Scored as either positive or negative (Nominal, dichotomous variable) [7]. |
| *Release test* | **Explanation:** From the final position of the relocation test, the tester abruptly stopped carrying out the centering of caput humeri.  **Placement of hands:** Removal of the tester’s wrist from the anterior part of the shoulder.  **Evaluation:** Subjective or objective reappearing apprehension symptoms and/or pain. Scored as either positive or negative (Nominal, dichotomous variable) [7]. |
| *Load and shift-test, anterior* | **Explanation:** The participant was supine with scapula resting on the examination table. Caput humeri was gently pushed into the joint socket through an axial pressure on the elbow. The shoulder was positioned in the scapula's plane in 90° abduction with the elbow flexed. The head of humerus was carefully pulled in an anterior direction.  **Placement of hands:** One hand the tester was placed on olecranon with the participant's hand positioned between the torso and elbow of the tester. The testers’ other hand was placed on top of the shoulder with the fingers on the back of the caput humeri, so that this could be moved/pulled anteriorly.  **Evaluation:** The translation of caput humeri was evaluated with a 4-level looseness-score. 0 = small, or almost no movement. 1 = caput humeri moved up and into glenoid. 2 = caput humeri moved farther than the glenoid but tracked back to the starting position when the pull was removed. 3 = caput humeri was moved beyond the glenoid and remained in this position, even if the pull was stopped. Scored as positive when the score was 2 or 3 (Ordinal variable) [7]. |
| *Load and shift-test, posterior* | **Explanation:** The participant was supine with scapula resting on the examination table. Caput humeri was gently pushed into the joint socket through an axial pressure on the elbow. The shoulder was placed in scaption with 20° abduction with the elbow slightly flexed. Caput humeri was now carefully moved in a posterior direction.  **Placement of hands:** One hand the tester was placed on olecranon with the participant's hand positioned between the torso and elbow of the tester. The tester’s wrist was placed anteriorly on the caput humeri, so it could be moved posteriorly by a push-movement.  **Evaluation:** The translation of caput humeri was evaluated with a 4-level looseness-score. 0 = small, or almost no movement. 1 = caput humeri moved up and into glenoid. 2 = caput humeri moved farther than the glenoid but tracked back to the starting position when the pull was removed. 3 = caput humeri was moved beyond the glenoid and remained in this position, even if the pull was stopped. Scored as positive when the score was 2 or 3 (Ordinal variable) [7]. |
| *Sulcus sign* | **Explanation:** The participant sat in an upright position. The tester was pulling the distal part of the humerus in a caudally direction. The distance between the top of the head of the humerus and acromion was measured with a ruler or measuring tape.  **Placement of hands:** A hand was placed over the epicondyle on humerus. The second hand was used to measure the subacromial distance between acromion and caput humeri, with a ruler.  **Evaluation:** Scored 1-3. 1: Less than 1 cm. 2: 1-2 cm. 3: Over 2 cm (Nominal, dichotomous variable) [7]. |
| *Gagey test* | **Explanation:** The participant sat in an upright position. The shoulder girdle was gently pressed in the depression direction with the arm moved passively into full horizontal abduction. A mirror in front of the participant was used to evaluate the shoulder abduction angle.  **Placement of hands:** The forearm was placed on top of the shoulder girdle with the other hand positioned round the elbow joint.  **Evaluation:** Scored as positive if the abduction was over 105 degrees (Nominal, dichotomous variable) [7]. |
| *Rotés Queról test* | **Explanation:** The participant sat in an upright position. The arm was moved actively in external rotation while holding the elbow to the body.  **Placement of hands:** A hand fixated processus coracoideus and acromion. The other hand had a grip on the forearm. External rotation was then started, and while being guided, the participant actively had to tried to externally rotate as far as possible.  **Evaluation:** Scored positive if external rotation was over 90 degrees (Nominal, dichotomous variable) [8]. |

**References**

1. Clarsen B, Bahr R, Andersson SH, Munk R, Myklebust G. Reduced glenohumeral rotation, external rotation weakness and scapular dyskinesis are risk factors for shoulder injuries among elite male handball players: a prospective cohort study. Br J Sports Med. 2014;48(17):1327-33.

2. Ingwersen KG, Christensen R, Sorensen L, Jorgensen HR, Jensen SL, Rasmussen S, Sogaard K, Juul-Kristensen B. Progressive high-load strength training compared with general low-load exercises in patients with rotator cuff tendinopathy: study protocol for a randomised controlled trial. Trials. 2015;16:27.

3. Kjaer BH, Magnusson SP, Warming S, Henriksen M, Krogsgaard MR, Juul-Kristensen B. Progressive early passive and active exercise therapy after surgical rotator cuff repair - study protocol for a randomized controlled trial (the CUT-N-MOVE trial). Trials. 2018;19(1):470.

4. Vafadar AK, Cote JN, Archambault PS. Interrater and Intrarater Reliability and Validity of 3 Measurement Methods for Shoulder-Position Sense. J Sport Rehabil. 2016;Technical Report 19:2014-0309.

5. Eshoj H, Rasmussen S, Frich LH, Hvass I, Christensen R, Jensen SL, Søndergaard J, Søgaard K, Juul-Kristensen B. A neuromuscular exercise programme versus standard care for patients with traumatic anterior shoulder instability: study protocol for a randomised controlled trial (the SINEX study). Trials. 2017;18(1):90-.

6. Nicholson LL, Chan C. The Upper Limb Hypermobility Assessment Tool: A novel validated measure of adult joint mobility. Musculoskeletal Science and Practice. 2018.

7. Eshoj H, Ingwersen KG, Larsen CM, Kjaer BH, Juul-Kristensen B. Intertester reliability of clinical shoulder instability and laxity tests in subjects with and without self-reported shoulder problems. BMJ Open. 2018;8(3):e018472.

8. Juul-Kristensen B, Rogind H, Jensen DV, Remvig L. Inter-examiner reproducibility of tests and criteria for generalized joint hypermobility and benign joint hypermobility syndrome. Rheumatology (Oxford). 2007;46(12):1835-41.
